# Bypass of Methoxyamine-Adducted Abasic Sites by Eukaryotic Translesion DNA Polymerases

**DOI:** 10.3390/ijms26020642

**Published:** 2025-01-14

**Authors:** Anna V. Yudkina, Anna A. Novikova, Anastasia D. Stolyarenko, Alena V. Makarova, Dmitry O. Zharkov

**Affiliations:** 1 Siberian Branch of the Russian Academy of Sciences Institute of Chemical Biology and Fundamental Medicine, 8 Lavrentieva Ave., 630090 Novosibirsk, Russia; 2 Department of Natural Sciences, Novosibirsk State University, 2 Pirogova St., 630090 Novosibirsk, Russia; 3 Institute of Gene Biology, Russian Academy of Sciences, 34/5 Vavilova St., 119334 Moscow, Russia

**Keywords:** DNA damage, methoxyamine, abasic sites, DNA polymerases, DNA replication, translesion synthesis

## Abstract

The apurinic/apyrimidinic site (AP site) is a highly mutagenic and cytotoxic DNA lesion. Normally, AP sites are removed from DNA by base excision repair (BER). Methoxyamine (MOX), a BER inhibitor currently under clinical trials as a tumor sensitizer, forms adducts with AP sites (AP-MOX) resistant to the key BER enzyme, AP endonuclease. As AP-MOX remains unrepaired, translesion DNA synthesis is expected to be the main mechanism of cellular response to this lesion. However, the mutagenic potential of AP-MOX is still unclear. Here, we compare the blocking and mutagenic properties of AP-MOX and the natural AP site for major eukaryotic DNA polymerases involved in translesion synthesis: DNA polymerases η, ι, ζ, Rev1, and primase–polymerase PrimPol. The miscoding properties of both abasic lesions remained mostly the same for each studied enzyme. In contrast, the blocking properties of AP-MOX compared to the AP site were DNA polymerase specific. Pol η and PrimPol bypassed both lesions with the same efficiency. The bypass of AP-MOX by Pol ι was 15-fold lower than that of the AP site. On the contrary, Rev1 bypassed AP-MOX 5-fold better than the AP site. Together, our data suggest that Rev1 is best suited to support synthesis across AP-MOX in human cells.

## 1. Introduction

Base loss by hydrolysis of the *N*-glycosidic bond in nucleotides leads to the formation of the most common and cytotoxic DNA lesions, apurinic/apyrimidinic sites (AP sites or abasic sites). AP sites form both spontaneously and enzymatically during cellular metabolism, for example, as intermediates of base excision repair (BER). Moreover, the rate of depurination increases under genotoxic stress. On average, the estimated background number of AP sites present in mammalian cells is ~0.1–1 per 10^6^ [1,2,3]. AP sites are also created by a number of xenobiotics, including anticancer agents.

AP sites, if they persist in DNA, are highly mutagenic and cytotoxic [4]. First of all, they block most DNA polymerases, producing single- and double-strand DNA breaks and, as a consequence, this leads to chromosomal aberrations and even cell death. Moreover, AP sites are non-instructive, and no error-free translesion synthesis across them is possible, which leads to base substitutions and frequent deletions [5,6,7,8,9,10,11,12]. The cytotoxicity and mutagenicity of AP sites find use in anticancer therapy. A number of oxidative and alkylating anticancer agents, such as bleomycin, temozolomide, pemetrexed, etc., directly or indirectly induce AP sites [13,14,15].

Despite many anticancer drugs initially working very efficiently, they often fail in the long term due to tumor resistance development [16]. DNA repair is one of the main sources of cancer cell resistance to therapy [17,18,19,20]. A broad spectrum of DNA damage produced by chemo- and radiotherapy is repaired by BER [16,21]. Therefore, the development of inhibitors of key players of the BER pathway for efficient anticancer treatment is of great interest [22,23,24,25]. One such BER inhibitor is methoxyamine (MOX, also known as TRC-102), which has recently been the subject of several clinical trials as a tumor sensitizer [26,27,28,29,30,31,32,33,34,35,36,37,38,39,40,41]. MOX reacts with AP sites and forms an adduct (AP-MOX) resistant to the key enzyme of the BER pathway, apurinic/apyrimidinic endonuclease 1 (APE1) [42,43,44]. Despite promising results of MOX clinical trials, the biological effects of this molecule have been poorly studied. Paradoxically, there is still no structure of the AP-MOX adduct, and the mechanism of APE1 inhibition and its mutagenic properties is not clear.

Low-fidelity DNA translesion synthesis is a mechanism of cell response to DNA damage that allows the DNA replication machinery to directly bypass DNA lesions. This process requires two DNA polymerases, an inserter and an extender. DNA polymerases of the Y-family Pol ι, Pol η, and Pol κ efficiently incorporate nucleotides opposite DNA lesions, while B-family DNA polymerase ζ extends DNA synthesis beyond the DNA damage site [45,46]. Another bifunctional Y-family DNA polymerase, Rev1, plays a major coordinating role by interacting with a Y-family polymerase, Pol ζ, and PCNA and accomplishing the switch between polymerases [47]. In some cases, Rev1 plays a catalytic role and incorporates dCMP opposite blocking DNA lesions, such as AP-site and 1,*N*^6^-ethenoadenine, as well as G4 quadruplexes [48,49,50,51]. Alternatively, in human cells, DNA primase and DNA polymerase PrimPol restart DNA replication at the sites of DNA damage [52]. Among all these enzymes functioning in humans, only Pol ζ, Pol η, and Rev1 are conserved throughout eukaryotes [45].

MOX might affect the activity of DNA replication enzymes during DNA synthesis across AP sites modulating their mutagenic potential and chemotherapy effectiveness. However, little is known about the effect of AP-MOX on the activity of human DNA polymerases. In our previous work [53], we demonstrated that the Klenow fragment of *Escherichia coli* DNA polymerase I, human DNA polymerases β and λ, and bacteriophage RB69 DNA polymerase bypassed AP-MOX less efficiently than natural AP sites, whereas human DNA polymerase κ was able to bypass AP-MOX more efficiently. Here, we studied the bypass and mutagenicity of AP-MOX on eukaryotic DNA polymerases, which are involved in DNA damage tolerance pathways, such as human DNA polymerases ι, η, Rev1, yeast DNA polymerase ζ, and human primase–polymerase PrimPol.

In this work, we show that AP-MOX maintains the mutagenic potential of the AP site as non-coding DNA damage. However, the blocking properties of AP-MOX substantially vary between different DNA polymerases. Together, our data suggest that Rev1 is best suited to bypass AP-MOX.

## 2. Results

### 2.1. General Experimental Design

To characterize the blocking and miscoding properties of the methoxyamine-conjugated AP site (AP-MOX) for eukaryotic translesion DNA polymerases, we used a standard primer extension assay to determine the preferable incorporated nucleotide opposite the damage. The lesion was placed in the +1 template position relative to the 3′-end of the labeled primer. If noticeable incorporation of an individual dNTP was achieved, steady-state kinetic parameters of nucleotide incorporation were measured and compared to those for the aldehyde AP site.

In this work, we tested human translesion DNA polymerases η, ι, and Rev1, belonging to the Y-family. Also, we used yeast DNA polymerase, ζ, which is highly similar to its human counterpart with respect to translesion synthesis [54]. Despite Pol ζ belonging to the B-family, it plays a crucial role in translesion synthesis by cooperating with other translesion DNA polymerases and enhancing their translesion activity [54]. Here, we tested the individual ability of DNA polymerases to bypass AP-MOX. The remaining representative of human translesion DNA polymerases, DNA polymerase κ, was a subject of our previous study [53].

### 2.2. Pol η Bypasses AP-MOX and the AP Site with the Same Efficiency

Pol η plays an important role in UV damage response, and its defects cause xeroderma pigmentosum variant syndrome [55]. Eukaryotic Pol η was suggested to be involved in error-prone synthesis across AP sites [56,57].

**Figure 1:**
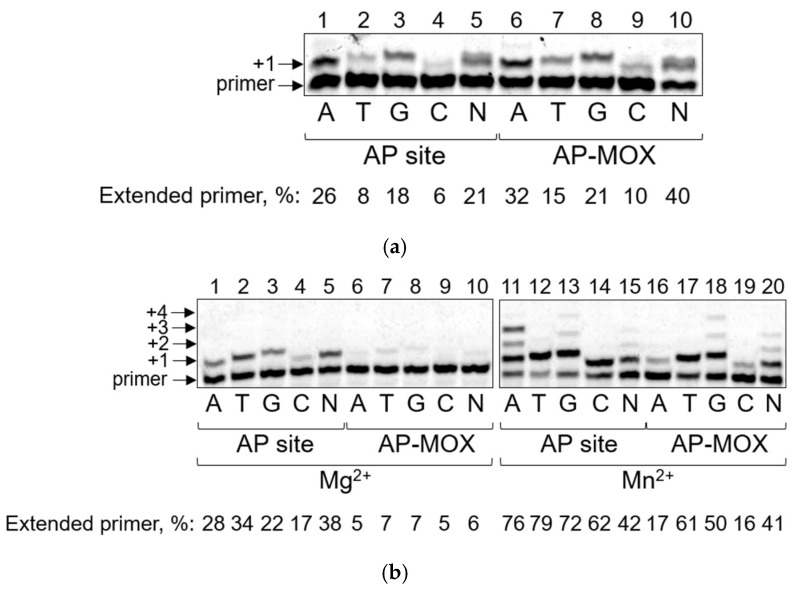
Nucleotide incorporation opposite the aldehydic AP site and AP-MOX by Pol η and Pol ι. (**a**) Synthesis by Pol η on the template containing an AP site (lanes 1–5) or AP-MOX (lanes 6–10). (**b**) Synthesis by Pol ι on the template containing an AP site (lanes 1–5, 11–15) or AP-MOX (lanes 6–10, 16–20) in the presence of Mg^2+^ (lanes 1–10) or Mn^2+^ (lanes 11–20). The nature of the cofactor, dNTPs (N, all four dNTPs in an equimolar ratio), and the percentage of the extended primer are indicated below the gel. Arrows mark the primer and the primer elongation products.

Expectedly, both non-coding lesions (the AP site and AP-MOX) significantly blocked the activity of Pol η. Since in our experiments we also visualize the template strand to confirm full conversion of the AP site to AP-MOX [53], we observed noticeable terminal-transferase activity of Pol η, comparable to nucleotide incorporation opposite the AP site or AP-MOX, which underlines strong blocking of this DNA polymerase by such lesions (Supplementary Figure S1). The blocking properties of AP-MOX were comparable with the aldehyde AP site, and the percent of nucleotide incorporation opposite the lesions was similar (Figure 1a). The spectrum of preferable incorporated nucleotides was consistent with the literature data that shows that Pol η prefers to incorporate dAMP or dGMP opposite the AP site in vitro [10,58,59]. This “A-rule” of nucleotide incorporation preference opposite the AP site is common for many DNA polymerases. The ability and preference of Pol η to incorporate both dAMP and dGMP were also observed in reactions with all four nucleotides by band doubling (Figure 1a, line 5, line 10). The observed incorporation preference for both the aldehyde AP site and AP-MOX was in the order A > G > T, C.

To quantify the miscoding properties of the lesions, we measured steady-state kinetic parameters of dNMP incorporation by DNA polymerases opposite AP-MOX in comparison with the enzymatically prepared aldehydic AP site (Table 1). The kinetic parameters confirmed that Pol η bypasses AP sites and AP-MOX with approximately the same efficiency and mutagenicity.

### 2.3. Pol ι Bypass of AP-MOX Is Significantly Decreased Compared to the AP Site

Pol ι is a paralog of Pol η [60]; however, the spectra of DNA lesions bypassed by Pol ι and Pol η are different [61]. It has been demonstrated that Pol ι efficiently incorporates one nucleotide opposite an AP site [62,63], and this activity is stimulated in the presence of Mn^2+^ [64]. Therefore, we tested the ability of Pol ι to bypass AP-MOX in the presence of either cofactor, Mg^2+^ or Mn^2+^.

Pol ι was indeed able to bypass the natural AP site very efficiently in the presence of Mg^2+^, incorporating all four dNMPs opposite the lesion (Figure 1b). Pol ι preferentially incorporated dTMP along with dAMP and dGMP. The high efficiency of dTMP incorporation opposite an AP site is atypical for most DNA polymerases. However, such Pol ι’s preference has been reported in the literature [62,63] and can be explained by a unique mechanism of Pol ι nucleotide incorporation [65]. While Pol ι bypassed the AP site efficiently, it demonstrated dramatically lower efficiency in bypassing AP-MOX in the presence of Mg^2+^. The incorporation of only trace amounts of dTMP and dGMP opposite AP-MOX was observed (Figure 1b).

Mn^2+^ greatly increased the translesion activity of Pol ι on both studied DNA substrates (Figure 1b). In the presence of Mn^2+^, Pol ι was able to bypass AP-MOX (but still less efficiently compared to the AP site) with the incorporation preference similar to the non-coding AP site: T > G > A >> C (see also [62,63]). Notably, along with the translesion activity, Mn^2+^ also stimulated the processivity of normally very-low-processive Pol ι.

Despite Pol ι being one of the most efficient DNA polymerases in the bypass of AP sites and many bulky adducts, it demonstrated the most significant drop in the bypass capability upon switching from the AP site to AP-MOX (15-fold decrease in reactions with Mn^2+^) (Table 2). The affinity of Pol ι (*K*_M_) for AP-MOX was reduced approximately two-fold compared to the AP site, while the rate of nucleotide incorporation opposite the damage (*k*_cat_) diminished approximately by an order of magnitude (Table 2).

### 2.4. Pol ζ and Rev1 Bypass AP-MOX More Efficiently than the AP Site

Eukaryotic Rev1 is a deoxycytidyl transferase specifically inserting dCMP opposite to G and some damaged nucleotides during translesion synthesis [48,49,50,66]. Pol ζ mostly participates in translesion synthesis extending an unpaired primer–template terminus [62]. Despite Pol ζ being crucial for TLS as an extender, it is capable of bypassing a variety of lesions in vitro, including AP sites [54,67]. It has been shown that the bypass of AP sites in vivo requires Rev1 in addition to Pol ζ [48,57,68].

**Figure 2:**
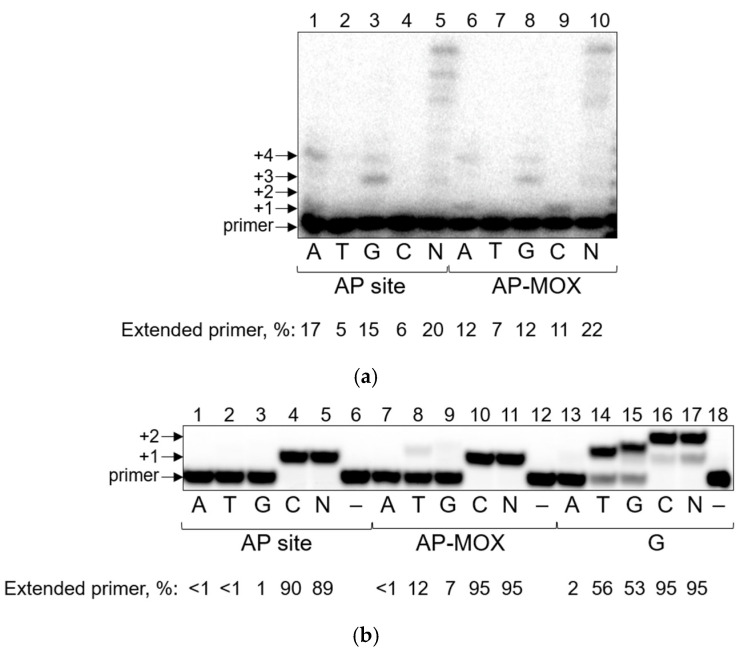
Nucleotide incorporation opposite the aldehydic AP site and AP-MOX by Pol ζ and Rev1. (**a**) Synthesis by Pol ζ on the template containing the AP site (lanes 1–5) or AP-MOX (lanes 6–10). (**b**) Synthesis by Rev1 on the template containing the AP site (lanes 1–6), AP-MOX (lanes 7–12), or G (lanes 13–18). The presence of dNTPs (N, all four dNTPs in an equimolar ratio) and the percentage of the extended primer are indicated below the gel. Arrows mark the primer and the primer elongation products.

Pol ζ demonstrated processive synthesis opposite the AP site and AP-MOX, even in the presence of individual dNTPs (Figure 2a). The efficiency and spectrum of nucleotide incorporation opposite AP-MOX were comparable with the natural AP site. The preference of incorporation was in the order A, G > C > T. However, a 4-fold increase in the incorporation of dCMP opposite AP-MOX compared to the AP site was detected in steady-state kinetic experiments (Table 3).

The AP site, along with G and its derivatives, is a canonical substrate for Rev1 [69]. Nucleotide incorporation opposite the AP site and AP-MOX was very efficient, even using a small concentration of the enzyme (Figure 2b). Interestingly, in the case of AP-MOX, the incorporation of trace amounts of dGMP and dTMP was observed (Figure 2b, lanes 8, 9) in contrast to the AP site. Such “non-complementary” (for deoxycytidyl transferase) incorporation was reported for G-containing substrates [69,70,71] (Figure 2b, lanes 14, 15).

Steady-state kinetic parameters demonstrated that despite the relatively higher efficiency of AP site bypass by Rev1 compared to the other DNA polymerases, AP-MOX bypass was further 5-fold more efficient than the bypass of a natural AP site (Table 4) but still 5-fold less efficient than the bypass of G. The incorporation of non-preferable dTMP and dGMP opposite AP-MOX was 400–2000-fold less efficient than the incorporation of dCMP, which is consistent with the literature data for the template G [71]. Our kinetic data obtained for template G agreed with these data. For the AP site, we did not see any noticeable incorporation of non-preferable dTMP and dGMP. These data may indicate that AP-MOX rather than the aldehydic AP site is a preferable substrate for Rev1.

Thus, we observed significantly increased activity of Rev1 on AP-MOX-containing substrates compared to the natural AP site. Pol ζ also demonstrated moderately increased activity of dCMP incorporation opposite AP-MOX compared to the AP site.

### 2.5. PrimPol Bypass of the AP Site and AP-MOX Is Stimulated by Mn^2+^

PrimPol is a unique primase–polymerase belonging to the archaeo-eukaryotic primase (AEP) superfamily [52]. PrimPol promotes replication fork progression by repriming DNA synthesis downstream of a lesion but also possesses DNA polymerase activity. It has been demonstrated that PrimPol efficiently incorporates nucleotides opposite a broad spectrum of lesions [72,73]. Moreover, it has been reported that Mn^2+^ ions significantly stimulate PrimPol’s polymerase activity, processivity, and ability to bypass lesions by increasing PrimPol’s affinity for substrates (both DNA and nucleotides) [74,75,76]. We performed primer extension assays for PrimPol in the presence of either Mg^2+^ or Mn^2+^.

**Figure 3:**
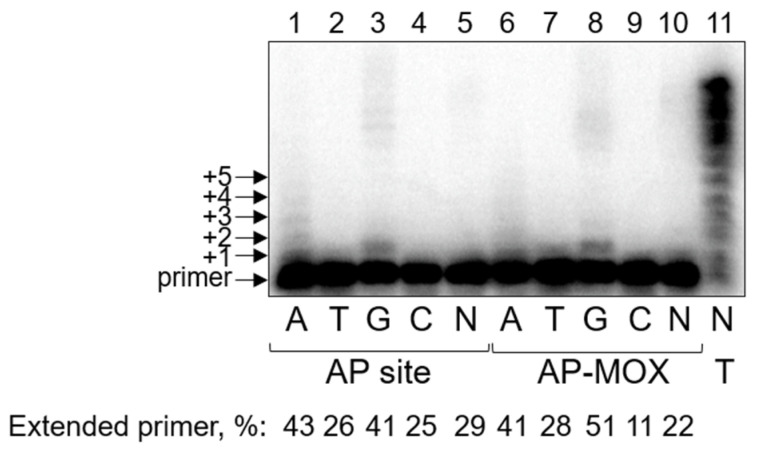
Nucleotide incorporation opposite the aldehydic AP site and AP-MOX by PrimPol. Synthesis on the template containing an AP site (lanes 1–5), AP-MOX (lanes 6–10), or T (lane 11) in the presence of Mn^2+^. The presence of dNTPs (N, all four dNTPs in an equimolar ratio) and the percentage of the extended primer are indicated below the gel. Arrows mark the primer and the primer elongation products.

Similarly to Pol ι, the presence of Mn^2+^ as a cofactor increases the processivity and translesion activity of PrimPol. In the presence of Mg^2+^, we observed no significant bypass of the AP site or AP-MOX (Supplementary Figure S2). However, cofactor replacement led to a noticeable increase in the translesion synthesis across the AP site and AP-MOX by PrimPol (Figure 3). The efficiency of nucleotide incorporation opposite the lesions seemed to be generally comparable for both substrates (Figure 3). PrimPol preferentially incorporated dAMP and dGMP on DNA substrates with the AP site and AP-MOX (A, G >> T, C). Even with individual dNMPs, the incorporation took place in a processive way, likely due to Mn^2+^ stimulation. These data suggest that PrimPol might follow the “A-rule” of nucleotide incorporation opposite both AP sites and AP-MOX. Alternatively, PrimPol might skip the lesion and incorporate dAMP and dGMP opposite templates C and T in positions +2…+4. The lesion-skipping (or template scrunching) activity of PrimPol is stimulated by Mn^2+^ ions and has been reported previously [72,76].

Steady-state kinetic parameters measured in the presence of Mn^2+^ indicated that PrimPol is blocked by AP-MOX somewhat more than the AP site (1.7–2.6-fold decrease in *k*_cat_/*K*_M_, Table 5). Interestingly, the rate of nucleotide incorporation opposite the damage was the same for both substrates, whereas the affinity of DNA polymerase for AP-MOX was decreased compared to the AP site (Table 5). The incorporation of dCMP and dTMP was too low for *K*_M_ and *k*_cat_ to be reliably estimated.

## 3. Discussion

**Figure 4:**
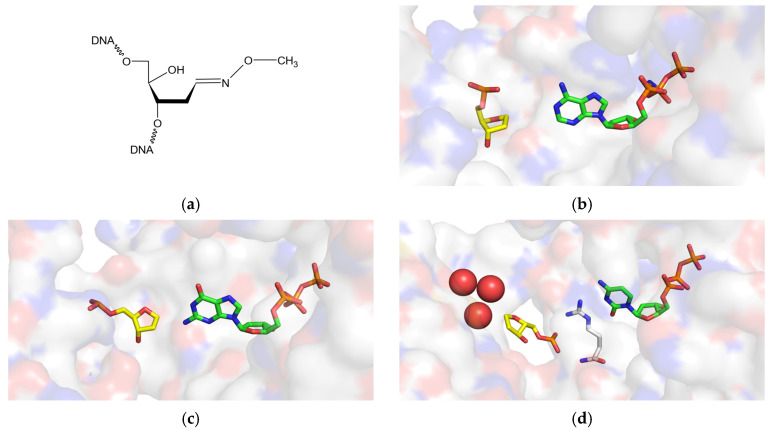
(**a**) Chemical structure of the AP-MOX adduct. (**b**–**d**) Active sites of ternary complexes of DNA polymerases binding an AP site and illustrating the orientation of the AP site (carbon atoms colored yellow) and the incoming dNTP (carbon atoms colored green) in the active site. Other atoms are color coded as follows: O, red, N, blue, P, orange. (**b**) Structure of Pol η binding an AP site (4RNM, [9]); (**c**) structure of Pol ι binding an AP site (3G6X, [65]); (**d**) structure of Rev1 binding an AP site (3OSP, [8]). Water molecules in the active site are shown as red balls.

Chemo- and radiotherapy still remain the main methods for cancer treatment. However, due to the systemic toxicity and the eventual emergence of resistance of cancer cells, the development of new strategies for treating tumors is necessary. One of the main reasons for cancer cells’ resistance to chemo- and radiotherapy is the upregulation of DNA repair mechanisms [16], where base excision repair plays the central role in removing the majority of oxidative and alkylation damage. Methoxyamine is the simplest aldehyde-reactive alkoxyamine which directly inhibits the base excision repair pathway. MOX reacts with the aldehyde group of the first common BER intermediate, the AP site (Figure 4a), preventing its cleavage by the AP endonuclease APE1 [77], one of the key enzymes of BER, which enhances the cytotoxicity of a wide range of anticancer agents. The results of several recently completed clinical trials indicate that a combination of MOX and temozolomide, an alkylating agent, is promising in the treatment of solid tumors, including glioblastomas, with poor prognosis [39,40,41]. Several other compounds reacting with AP sites through similar chemistry but are more selective due to the presence of well-stacking moieties, such as naphthalenophanes, anthraquinones, and phthalazines, are now under investigation as tumor cell sensitizers [78,79,80,81]. MOX also dampens the expression of pro-inflammatory cytokines in an APE1-dependent manner [82].

An AP site, if unrepaired, is a target for translesion synthesis by DNA polymerases. However, an AP site is one of the deadliest DNA lesions for the cell because of its highly blocking and mutagenic properties due to the inability to form complementary bonds with incoming nucleotides [4]. Moreover, due to the reactivity of AP sites, they easily yield a number of derivatives (for example, oxidized AP sites, adducts of AP sites with proteins, peptides, low-molecular-weight compounds, etc.). Importantly, a modification of an AP site, even small in size, may dramatically change the properties of the lesion and its biological effects [83,84]. This should be kept in mind when comparing our results on the natural AP site with the bulk of the literature data obtained with the tetrahydrofuran AP site analog, which in some cases behaves differently when compared to the natural AP site [83,84].

The main challenge for translesion synthesis by DNA polymerases across AP sites and their derivatives is the non-coding properties of these lesions. DNA polymerases are commonly blocked by non-instructive nucleotides to a greater extent than by bulky adducts. However, in contrast to a chemically labile AP site, which exists in an equilibrium between the minor open aldehyde and the predominant closed furanose forms and is easily converted to a strand break, AP-MOX is always open and much more stable [53]. Thus, we suggested that the conjugation of MOX to the AP site might change the ability of DNA polymerases to carry out translesion synthesis.

The interaction of human translesion DNA polymerases with AP-MOX conjugates has been barely addressed so far. Recently, we studied the blocking and miscoding properties of AP-MOX for several DNA polymerases of different species: the Klenow fragment of *E. coli* DNA polymerase I, phage RB69 DNA polymerase, and human DNA polymerases β, λ, and κ. Interestingly, only translesion Pol κ of family Y bypassed AP-MOX 5-fold more efficiently than the AP site, whereas the other DNA polymerases of the A, B, and X families bypassed AP-MOX less efficiently [53]. Therefore, in the present work, we have addressed the ability of the remaining eukaryotic translesion DNA polymerases, which are most likely to be involved in the bypass of AP-MOX in human cells, to incorporate nucleotides opposite to AP-MOX.

Although it is believed that the main function of Pol η is replication of UV-damaged DNA [85], the contribution of Pol η to AP site bypass in human cells is still unclear. It has been reported that Pol η is appreciably blocked by the AP site in vitro; the enzyme exhibits moderate nucleotide incorporation opposite the non-coding damage and extends further [7,10,59]. However, in yeast cells, it has been demonstrated that Pol η makes a significant contribution to AP site bypass [57]. Indeed, compared to other DNA polymerases, Pol η demonstrates relatively good efficiency in the synthesis across the AP site. In our work, we have shown that Pol η bypasses the AP site and AP-MOX with the same efficiency and mutagenicity under the standing-start conditions. The preference of Pol η to incorporate dAMP and dGMP opposite the AP site can be explained by hydrogen bonds forming between the 5′ phosphate of the AP site, a water molecule, and the incoming purine (N^6^ of dAMP or O^6^ and N1 of dGMP) [9]. Although there is still no three-dimensional structure of the MOX-conjugated AP site, the chemical structure of the adduct (Figure 4a) permits forming these interactions (Figure 4b). Indeed, in agreement with the structural expectations, our kinetic parameters of nucleotide incorporation opposite AP-MOX were the same as for the AP site. The contribution of Pol η to bypassing AP-MOX in vivo is yet to be established.

Pol ι also carries out efficient translesion synthesis across the AP site [62,63]. Moreover, the relative efficiency of the incorporation of all four nucleotides opposite the AP site was comparable or superior to complementary dCTP incorporation opposite undamaged G [7]. Indeed, we found good incorporation efficiency opposite the AP site in the presence of both Mg^2+^ and Mn^2+^. However, the efficiency of the AP-MOX bypass dropped 15-fold compared to the AP site, the strongest degree of inhibition among the studied DNA polymerases. In contrast to many DNA polymerase active sites, the AP site in the Pol ι active site does not loop or bulge out of the helix [65]. On the contrary, the abasic sugar is accommodated and fixed intrahelically opposite the incoming dNTP in the quite tight active site [65] (Figure 4c). The AP site is tucked into a small hydrophobic pocket formed by aliphatic and bulky residues Gln59, Lys60, Tyr61, and Leu62, which shorten the C1′-C1′ distance in the AP:dNTP pair. Thus, the presence of MOX conjugated with the AP site may structurally interfere with the correct positioning of the incoming dNTP in the active site of Pol ι. Indeed, our kinetic data show that *k*_cat_ but not *K*_M_ is affected when Pol ι accommodates and incorporates the incoming dNTP opposite AP-MOX (Table 2), suggesting that the catalytic step of the reaction rather than dNTP binding is hindered. Therefore, Pol ι is unlikely to participate in the AP-MOX bypass in vivo.

PrimPol possesses translesion synthesis activity, but the role of PrimPol in lesion bypass in vivo is not well established. Nevertheless, PrimPol can encounter AP-MOX during DNA repriming events. We demonstrated PrimPol’s preference to incorporate dAMP and dGMP in steady-state kinetic experiments. However, this preference could be explained not only by incorporation opposite the AP site and AP-MOX but also by template slippage. It has been shown that PrimPol tends to produce deletion by “skipping” damaged template nucleotides, and this activity is enhanced in the presence of Mn^2+^ [76]. In our experiments, we used the template strand containing C in the +1 position downstream of the damaged site and T in the +2 and +3 positions, which would direct the incorporation of dGMP and dAMP, respectively. Therefore, the observed incorporation presently cannot be ascribed to the true misincorporation or misalignment event; this matter requires further investigation. However, we can conclude that these events, whatever their nature is, happen with comparable efficiency for the AP site and AP-MOX. Another possibility is that PrimPol could augment AP-MOX bypass by other polymerases, as was shown for Pol ζ on cisplatin cross-links [86].

It is believed that the bypass of an unrepaired AP site in eukaryotes is carried out jointly by Rev1 and Pol ζ [48,57,68]. Rev1 bypasses an AP site very efficiently and is as “error-free” as possible with a non-coding lesion. Because *N*-glycosidic bond hydrolysis most easily occurs in guanine deoxynucleotides [87], the incorporation of dCMP by Rev1 can minimize the mutagenic potential of the damage. The Rev1 active site is remarkably adapted for AP site bypass. The structure of the active site, accommodating the AP site, is generally the same as that accommodating template G [8] (Figure 4d). The lesion is located extrahelically, the incoming dCMP is recognized by an Arg residue, and the hydrophobic cavity (that normally accommodates the unmodified G) is filled with three water molecules in the case of an AP site [8] (Figure 4d). Two of these waters correspond to O^6^ and N7 atoms of guanine. Nevertheless, in our experiments, the efficiency of the AP site bypass was approximately 30-fold lower than that of undamaged G, which is consistent with the literature data [7,70,88,89,90,91]. The drop in the total kinetic efficiency is due to *K*_M_ rather than *k*_cat_, implying that the architecture of the Rev1 active site, accommodating G and the AP site, is identical, but the affinity for the incoming dNTP is lower. In the case of AP-MOX bypass, we expect the same catalytic architecture of the active site. Steady-state kinetic parameters confirmed this hypothesis and demonstrated the same *k*_cat_ for AP-MOX, the AP site, and G. Interestingly, *K*_M_ for AP-MOX was 4–5-fold higher than for the AP site. We suggest that when AP-MOX is bound in Rev1’s hydrophobic cavity, it partly fills the space normally occupied by guanine and expels some of the water molecules present in the case of the AP site [8]. The nitrogen of MOX may replace a water molecule in the cavity or stabilize a water molecule by hydrogen bonding, which would decrease the binding entropy and thus increase the affinity of Rev1 for the substrate. Therefore, the active site of Rev1 is more suitable for accommodating modified AP sites, in particular AP-MOX. Our results highlight the need for further investigation of how Rev1 bypasses such types of damage and whether it has a role in their bypass in the cell.

Our data also demonstrated that Pol ζ is able to bypass AP-MOX slightly more efficiently than the intact AP site. Nevertheless, given Rev1’s efficiency as an inserter polymerase, it is more likely that the bypass of AP-MOX involves Pol ζ as an extender, elongating DNA downstream of the damage site after dCMP insertion by Rev1.

**Figure 5:**
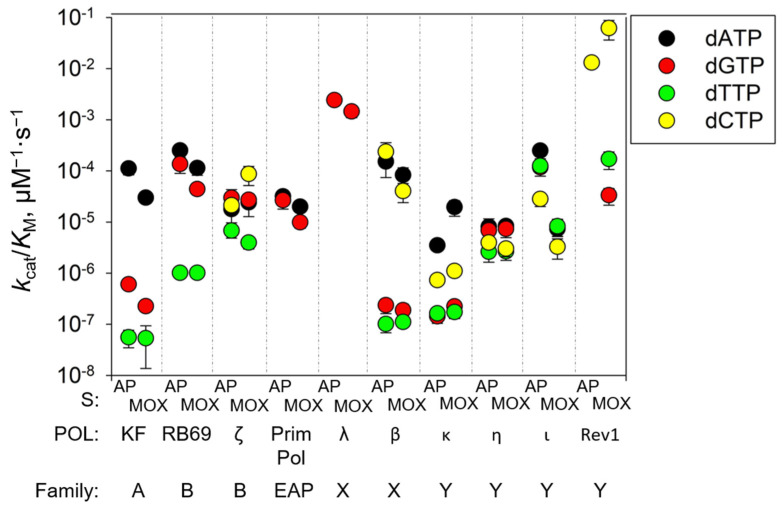
Graphical summary of the kinetics for all DNA polymerases studied in this work and in [53], showing the comparative efficiency and fidelity of the AP site and AP-MOX bypass.

The comparative efficiency and fidelity of the AP site and AP-MOX bypass by DNA polymerases studied here and in our recent work [53] are summarized in Figure 5. Altogether, our data showed that DNA polymerases of the Y-family (except Pol ι) are relatively efficient opposite AP-MOX and suggest that the pair of DNA polymerases, Rev1/Pol ζ, is best suited for efficient translesion synthesis across AP-MOX in vivo. This branch of the translesion pathway can decrease the mutagenic potential of MOX in cells. Moreover, the efficiency of translesion synthesis by Y-family DNA polymerases might be increased by the presence of Rev1 and PCNA; the known examples of such synergistic action include the stimulation of Pol ι bypass of [6→4]T-T photoproducts and AP sites by PCNA [92], the stimulation of the Pol κ bypass of UV-induced lesions and benz[*a*]pyrene adducts by Rev1 [93], etc. (reviewed in [94]).

Despite the fact that our studies are inherently limited by their in vitro nature, they provide an important insight into the prospect of the therapeutic use of MOX or its analogs with the same mechanism of action. In order to assess the risk of side effects caused by MOX, it is important to know the cytotoxic and mutagenic potential of AP-MOX adducts. From the polymerase data obtained so far, it seems that they are similar to the properties of the natural AP sites, and, therefore, the capacity to repair AP-MOX through non-BER pathways would be critical for cell survival upon combined treatment by MOX and DNA-damaging drugs. For example, cells deficient in the recombination repair pathway are hypersensitive to treatments that induce AP sites or suppress their repair [95,96,97]. Such selective DNA repair insufficiency is often observed in cancer cells, and alternative repair pathways can be targeted to sensitize them to genotoxic agents of intrinsic metabolic genotoxic stress while leaving normal cells relatively unaffected. Efficient translesion synthesis across AP-MOX may serve as one of the protective mechanisms. The possibility of resistance to AP-MOX adducts through homologous recombination and nucleotide excision repair is worth close attention.

## 4. Materials and Methods

### 4.1. Enzymes and Oligonucleotides

Human primase–polymerase PrimPol [86], human DNA polymerase η (Pol η) [86,98], human DNA polymerase ι (Pol ι) [99], yeast DNA polymerase ζ (Pol ζ) [100], and human deoxycytidyl transferase Rev1 [69] were overexpressed and purified as described. *E. coli* uracil–DNA glycosylase (Ung) and individual dNTPs were purchased from SibEnzyme (Novosibirsk, Russia). Methoxyamine was purchased from MP Biomedicals (Santa Ana, CA, USA). Oligonucleotides (template, 5′-CTCTCCCTTCUCTCCTTTCCTCT-3′, primer, 5′-AGAGGAAAGGAG-3′) were synthesized in-house from commercially available phosphoramidites (Glen Research, Sterling, VA, USA). The template oligonucleotide was 5′-FAM, labeled to confirm the AP site and MOX adduct formation; the primer oligonucleotide was 5′-FAM, labeled or ^32^P-labeled if necessary. DNA substrates containing the aldehyde AP site or MOX-AP were prepared from the template oligonucleotide, as described in [53].

### 4.2. Standing-Start Assay

The reaction mixture (10 μL) contained 100 nM preannealed oligonucleotide substrate, DNA polymerase in the appropriate buffer, and dNTPs (A, C, G, T, or a mixture of each). The buffers, concentrations of enzymes, and dNTPs were optimized for each DNA polymerase (Supplementary Table S1). The reaction was allowed to proceed for 30 min at 25 °C (or 10 min at 37 °C for Rev1) and stopped by adding an equal volume of 10 mM EDTA in formamide and heating at 95 °C for 2 min. Reaction products were resolved by electrophoresis in 20% polyacrylamide gels with 8 M urea and visualized and quantified using Typhoon FLA 9400 (GE Healthcare Inc., Chicago, IL, USA) and Quantity One v.4.6.3 software (Bio-Rad Laboratories, Hercules, CA, USA). All experiments were repeated at least two times. The percent of the product was calculated as a percent of all bands corresponding to extended primer for each individual reaction.

### 4.3. Steady-State Kinetics

The reaction mixture (10 μL) contained 100 nM preannealed oligonucleotide substrate, DNA polymerase in the appropriate buffer (Supplementary Table S1), and 0.5–1000 μM dNTPs (A, T, G, or C). The concentration of each DNA polymerase was optimized to incorporate less than 20% of the first dNMP in 30 min at 25 °C (or 5 min at 37 °C for Rev1), and the concentrations for all DNA polymerases are shown in Supplementary Table S1. The reactions were allowed to proceed for 30 min at 25 °C (or 5 min at 37 °C for Rev1), and they were then terminated and processed as described above. The data were fitted to the Michaelis–Menten equation by nonlinear regression using SigmaPlot v11.0 (Systat Software, Chicago, IL, USA). All reported constants are derived from three to five independent experiments.

## Figures and Tables

**Table 1:** Steady-state kinetic parameters for dNMP incorporation opposite the aldehydic AP site and AP-MOX by Pol η (mean ± s.e.).

Pol	Template	dNTP	*K*_M_, µM	*k*_cat_, s^−1^ (×10^5^)	*k*_cat_/*K*_M_, µM^−1^s^−1^ (×10^6^)
Pol η	AP site	dATP	104 ± 40	84 ± 14	8.1 ± 3.4
Pol η	AP site	dGTP	116 ± 39	79 ± 9	6.8 ± 2.4
Pol η	AP site	dTTP	197 ± 69	52 ± 7	2.6 ± 1.0
Pol η	AP site	dCTP	127 ± 26	50 ± 5	4.0 ± 0.9
Pol η	AP-MOX	dATP	130 ± 27	108 ± 9	8.3 ± 1.8
Pol η	AP-MOX	dGTP	102 ± 32	75 ± 8	7.3 ± 2.4
Pol η	AP-MOX	dTTP	213 ± 67	57 ± 7	2.7 ± 0.9
Pol η	AP-MOX	dCTP	154 ± 45	47 ± 5	3.0 ± 0.9

**Table 2:** Steady-state kinetic parameters for dNMP incorporation opposite the aldehydic AP site and AP-MOX in the presence of Mn^2+^ by Pol ι (mean ± s.e.).

Pol	Template	dNTP	*K*_M_, µM	*k*_cat_, s^−1^ (×10^5^)	*k*_cat_/*K*_M_, µM^−1^s^−1^ (×10^6^)
Pol ι	AP site	dATP	2.3 ± 0.7	57 ± 3	248 ± 75
Pol ι	AP site	dGTP	2.6 ± 0.8	30 ± 2	117 ± 39
Pol ι	AP site	dTTP	2.1 ± 0.5	27 ± 1	125 ± 33
Pol ι	AP site	dCTP	5.1 ± 1.5	14 ± 0.8	28 ± 8.2
Pol ι	AP-MOX	dATP	5.0 ± 1.4	3.7 ± 0.3	7.4 ± 2.1
Pol ι	AP-MOX	dGTP	5.4 ± 1.9	4.5 ± 0.4	8.2 ± 3.0
Pol ι	AP-MOX	dTTP	4.2 ± 1.5	3.5 ± 0.3	8.2 ± 3.1
Pol ι	AP-MOX	dCTP	3.7 ± 1.5	1.2 ± 0.1	3.3 ± 1.4

**Table 3:** Steady-state kinetic parameters for dNMP incorporation opposite the aldehydic AP site and AP-MOX by Pol ζ (mean ± s.e.).

Pol	Template	dNTP	*K*_M_, µM	*k*_cat_, s^−1^ (×10^5^)	*k*_cat_/*K*_M_, µM^−1^s^−1^ (×10^6^)
Pol ζ	AP site	dATP	79 ± 35	141 ± 16	18 ± 8
Pol ζ	AP site	dGTP	32 ± 14	96 ± 9	30 ± 13
Pol ζ	AP site	dTTP	21 ± 6	15 ± 1	7 ± 2
Pol ζ	AP site	dCTP	14 ± 4	31 ± 2	21 ± 7
Pol ζ	AP-MOX	dATP	32 ± 15	78 ± 9	24 ± 11
Pol ζ	AP-MOX	dGTP	23 ± 7	63 ± 4	27 ± 8
Pol ζ	AP-MOX	dTTP	35 ± 9	14 ± 1	4 ± 1
Pol ζ	AP-MOX	dCTP	5 ± 2	41 ± 3	87 ± 35

**Table 4:** Steady-state kinetic parameters for dNMP incorporation opposite the aldehydic AP site and AP-MOX by Rev1 (mean ± s.e.).

Pol	Template	dNTP	*K*_M_, µM	*k*_cat_, s^−1^ (×10^2^)	*k*_cat_/*K*_M_, µM^−1^s^−1^ (×10^3^)
Rev1	AP site	dCTP	21 ± 4	27 ± 1	13 ± 2
Rev1	AP site	dTTP	no incorporation		
Rev1	AP site	dGTP	no incorporation		
Rev1	AP-MOX	dCTP	4.7 ± 1.9	29 ± 3	62 ± 26
Rev1	AP-MOX	dTTP	108 ± 39	1.9 ± 0.2	0.17 ± 0.06
Rev1	AP-MOX	dGTP	169 ± 57	0.6 ± 0.1	0.03 ± 0.01
Rev1	G	dCTP	0.7 ± 0.1	25 ± 1	340 ± 50
Rev1	G	dTTP	86 ± 24	3.0 ± 0.3	0.35 ± 0.10
Rev1	G	dGTP	87 ± 26	3.1 ± 0.3	0.36 ± 0.11

**Table 5:** Steady-state kinetic parameters for dNMP incorporation opposite the aldehydic AP site and AP-MOX in the presence of Mn^2+^ by PrimPol (mean ± s.e.).

Pol	Template	dNTP	*K*_M_, µM	*k*_cat_, s^−1^ (×10^6^)	*k*_cat_/*K*_M_, µM^−1^s^−1^ (×10^6^)
PrimPol	AP site	dATP	1.1 ± 0.3	35 ± 13	32 ± 9
PrimPol	AP site	dGTP	1.1 ± 0.4	30 ± 13	27 ± 19
PrimPol	AP-MOX	dATP	1.8 ± 0.4	35 ± 10	20 ± 4
PrimPol	AP-MOX	dGTP	3.7 ± 0.8	36 ± 13	10 ± 2

## Data Availability

All data are contained in the paper.

## Supplementary Materials

The following supporting information can be downloaded at https://www.mdpi.com/article/10.3390/ijms26020642/s1.
