# Supplementary material for: Bypass of Methoxyamine-Adducted Abasic Sites by Eukaryotic Translesion DNA Polymerases
Source: Int J Mol Sci. 2025 Jan 14;26(2):642. doi: 10.3390/ijms26020642 (PMC11766430; doi:10.3390/ijms26020642)
Supplement: Supplementary file 1 [file ijms-26-00642-s001.zip › ijms-3402661-supplementary.pdf]

Supplementary Materials

Supplementary Table S1. Experimental conditions and composition of buffers used in this work.

**Standing start assay**					
	POLι	POLη	POLζ	PrimPOL	Rev1
5′ label of primer	FAM, ^{32}P	FAM, ^{32}P	^{32}P	^{32}P	FAM
DNA polymerase concentration, nM	25	50	40	400	1
dNTP concentration, μM	50	50	50	250	200
**Steady-State Kinetics assay**					
5′ label of primer	FAM	FAM	FAM	^{32}P	FAM
DNA polymerase concentration, nM	15–1000	20	10–20	400	0.2–20
dNTP concentration range, μM	2–250	1–1000	2–1000	1–500	0.2–750
**Appropriate buffer composition**					
Buffer composition 1×	Mg^{2+} buffer: 30 mM HEPES pH 7.4, 50 mM NaCl, 0.1 mg/ml BSA, 1 mM $MgCl_2$, 1 mM DTT or Mn^{2+} buffer: 30 mM HEPES pH 7.4, 50 mM NaCl, 0.1 mg/ml BSA, 0.5 mM $MnCl_2$, 1 mM DTT	30 mM HEPES pH 7.4, 50 mM NaCl, 0.1 mg/ml BSA, 10 mM $MgCl_2$, 1 mM DTT	30 mM HEPES pH 7.4, 50 mM NaCl, 0.1 mg/ml BSA, 10 mM $MgCl_2$, 1 mM DTT	Mg^{2+} buffer: 30 mM HEPES pH 7.2, 5% glycerin, 0.1 mg/ml BSA, 10 mM $MgCl_2$, 1 mM DTT or Mn^{2+} buffer: 30 mM HEPES pH 7.2, 5% glycerin, 0.1 mg/ml BSA, 0.5 mM $MnCl_2$, 1 mM DTT	30 mM HEPES pH 7.6, 50 mM NaCl, 0.1 mg/ml BSA, 10 mM $MgCl_2$, 1 mM DTT

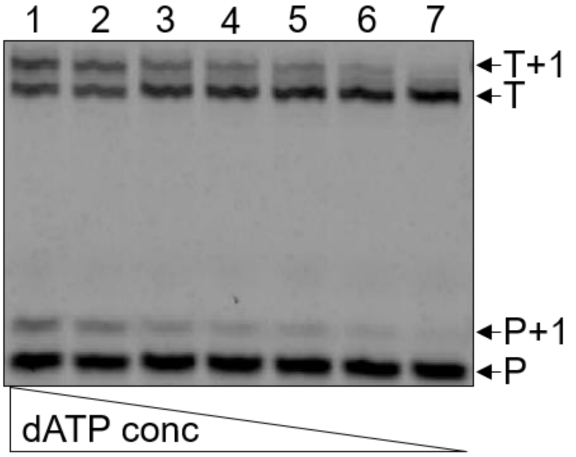

Supplementary Figure S1. Steady-state kinetics reactions of Pol η's synthesis on the AP-site containing substrate in the presence of different concentrations of dATP. P – primer; P+1 – extended primer during polymerase reaction; T – FAM-labeled template strand to visualize the completeness of AP site formation by Ung; T+1 – presumably terminal transferase activity of Pol η.

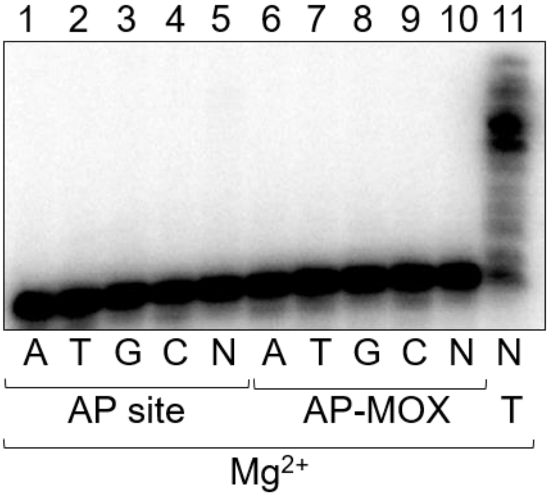

Supplementary Figure S2. Nucleotide incorporation opposite the aldehyde AP site and AP-MOX by PrimPol in the presence of Mg^{2+}. Synthesis on the template containing the AP site (lanes 1–5), AP-MOX (lanes 6–10) and T (lane 11). The presence of dNTPs (N, all four dNTP in an equimolar ratio) is indicated below the gel.
